# Effect of the intermittent Pringle maneuver on liver damage after hepatectomy: a retrospective cohort study

**DOI:** 10.1186/s12957-019-1680-y

**Published:** 2019-08-13

**Authors:** Xiaolin Wei, Wenjing Zheng, Zhiqing Yang, Hui Liu, Tengqian Tang, Xiaowu Li, Xiangde Liu

**Affiliations:** 1 Shenzhen University General Hospital & Shenzhen University Clinical Medical Academy, No. 1098, Xue Yuan Avenue, Xili University Town, Shenzhen, 518055 Guangdong China; 2 Southwest Hospital, Third Military Medical University (Army Medical University), No. 30, Gaotanyan Street, Shapingba District, Chongqing, 400038 China

**Keywords:** Hepatectomy, Pringle maneuver, Liver damage, Retrospective.

## Abstract

**Background:**

The Pringle maneuver (PM) interrupts the blood flow through the hepatic artery and portal vein to help control bleeding. This study analyzes the effects of the intermittent Pringle maneuver (IPM) on the surgical process and postoperative liver injury.

**Methods:**

This study retrospectively evaluated 182 hepatocellular carcinoma patients who underwent hepatectomy. In the IPM group, hepatic blood flow was intermittently interrupted via clamping, with cycles of 10 minutes of inflow occlusion followed by 5 minutes of reperfusion that were repeated until the end of the surgery. In the non-IPM group, liver resection was performed without hepatic vascular blockage.

**Results:**

For postoperative complications, the incidence rates of ascites and pleural effusion in the IPM group were significantly lower than those in the non-IPM group. The postoperative hospitalization time in the IPM group was significantly lower than that in the non-IPM group (*p*=0.0008). On the first day after the operation, the platelet count was significantly lower (*p*=0.0381) but the prothrombin time (PT) (*p*=0.0195) and activated partial thromboplastin time (APTT) (*p*=0.0071) were significantly higher in the non-IPM group than those in the IPM group. At discharge, only albumin was significantly higher in the non-IPM group than that in the IPM group (*p*=0.0303). Regression analysis showed that a prolonged interruption time was related to increased ALT and AST levels on the first day after surgery, but not on the seventh day or at discharge.

**Conclusion:**

The IPM does not cause additional liver damage during hepatectomy, and use of the IPM results in shorter hospital stays compared to surgery without using the IPM. The results of this study require further confirmation because of the retrospective design.

**Electronic supplementary material:**

The online version of this article (10.1186/s12957-019-1680-y) contains supplementary material, which is available to authorized users.

## Introduction

Hepatectomy is the most effective method to treat hepatobiliary cancer, such as hepatic carcinoma. Massive bleeding is usually the major problem in hepatectomy. Although successful hepatectomy does not necessarily require blocking hepatic blood flow [1], controlling the hepatic blood flow is helpful for maintaining a relatively bloodless surgical environment, disconnecting the liver, reducing intraoperative bleeding, and shortening the operation time. The Pringle maneuver (PM) is a surgical maneuver used to interrupt the blood flow through the hepatic artery and portal vein to help control bleeding from the liver; the PM is technically easy to implement and frequently used by surgeons [2].

However, the advantages and disadvantages of the PM remain controversial [3, 4]. Unlike the effect of the PM on liver dysfunction in animal experiments, in clinical practice, although blocking hepatic blood flow leads to hepatic ischemia, metabolism in the human liver is not significantly affected [5, 6]. The main reason may be the more abundant collateral circulation in the human liver than that in the livers of animal models. In addition, the tolerance of the liver to warm ischemia and ischemia-reperfusion injury induced by the PM may be related to the duration of hepatic ischemia [7]. An intermittent PM (IPM) can partially reduce ischemic damage to the residual liver, thus prolonging the total tolerable time of the residual liver to ischemia.

The ability of the residual liver to regenerate is another important aspect of evaluating the success of hepatectomy, and the effect of intraoperative hepatic blood flow occlusion on liver regeneration remains controversial [8]. Thermal ischemia of the liver may lead to protein synthesis dysfunction in hepatocytes. However, a study has shown that the PM does not affect liver regeneration after hepatectomy, and short-term thermal ischemia can even accelerate liver regeneration [9]. In this study, we retrospectively analyzed the effects of the IPM on the surgical process and recovery from postoperative liver injury and compared hepatectomy with the IPM to hepatectomy without the IPM.

## Patients and methods

### Patients

This study retrospectively evaluated 182 hepatocellular carcinoma patients who underwent open hepatectomy in the hepatological surgery department of our hospital from 2012 to 2016. The patient age range was 21 to 84 years old, with 150 males and 32 females. In total, 108 patients were included in the IPM group, and 74 patients were included in the non-IPM group. The inclusion criteria were as follows: age ≥ 18 years and hepatocellular carcinoma requiring hepatectomy. The following patients were excluded from this study: patients who previously underwent major operations on the liver or adjacent areas and patients who did not undergo liver resection. All included patients were consecutive patients who met the inclusion criteria. This study was performed in accordance with the Declaration of Helsinki and was approved by the Ethics Committee of Southwest Hospital, Third Military Medical University (Army Medical University).

### Preoperative evaluation

The gender, age, and clinical diagnosis of each patient were recorded before the operation. Liver-related complications and other comorbidities were also recorded. Preoperative laboratory blood tests included alanine aminotransferase (ALT), aspartate aminotransferase (AST), serum albumin, total bilirubin, hepatitis B surface antigen (HBsAg), and alpha-fetoprotein (AFP) levels, the presence of hepatitis B virus (HBV) deoxyribonucleic acid (DNA), the platelet count, and the prothrombin time (PT). The Child-Pugh classification scheme was used to assess the liver reserve function of the patients [10]. For patients with hepatic carcinoma, tumor, node, metastasis (TNM) staging was evaluated.

### Surgical procedure

All surgical procedures were performed by our departmental doctors to ensure consistency. The extent of liver resection was determined by precise segmental resection. The hepatoduodenal ligament was clamped to control the hepatic vasculature until the hepatic artery pulse disappeared distally. In the IPM group, the hepatic vasculature was intermittently clamped, with cycles of 10 min of inflow occlusion followed by 5 min of reperfusion that were repeated until the end of the surgery (additional illustrations and movie files show this in more detail see Additional file 1 and Additional file 2). In the non-IPM group, liver resection was performed without hepatic blockage. The duration of hepatic vascular occlusion (excluding the open period), the number of occlusions, the duration of the operation, and the amount of bleeding during the operation were recorded.


Additional file 2:Video of surgical procedure. (FLV 15850 kb)


### Postoperative management

Postoperative complications and the durations of hospital and intensive care unit (ICU) stays were recorded. The leukocyte count, neutrophil ratio, platelet count, ALT, AST, serum albumin, total bilirubin, and D-dimer (D-D) levels, PT, and activated partial thromboplastin time (APTT) were measured on the first, third, fifth, and seventh postoperative days and at discharge. No results were recorded when the test was normal or the patient refused the test.

### Statistical analysis

The qualitative data are expressed as frequencies (percentages), and statistical significance was evaluated using the *χ*^2^ test. Quantitative data are expressed as the mean ± standard deviation, and the groups were compared using analysis of variance (ANOVA) if the data were normally distributed as detected by skewness and kurtosis tests. Non-normally distributed data were compared with the Kruskal-Wallis test and are expressed as the medians and quartiles. The *p* value in multiple analyses was corrected by false discovery rate (FDR) methods. Linear regression analysis was used to analyze the interruption time and postoperative liver injury. Recovery of liver function with increasing postoperative time in IPM patients and non-IPM patients was further analyzed. This seemingly unrelated estimation was used to test the difference in regression equation coefficients [11]. All the calculations were performed with STATA 14.0 software (StataCorp LLC, TX, US), and results with *p* < 0.05 were considered significant.

## Results

No difference in the mean age or sex ratio was found between the IPM and non-IPM groups. No significant differences were identified between the two groups in terms of hepatic comorbidities. No significant differences in the rates of hypertension and diabetes were noted between the two groups. With regard to the laboratory tests, no significant differences in the ALT, AST, albumin, total bilirubin, HBsAg, or AFP levels, PT, platelet count, or amount of HBV DNA were found between the two groups (Table 1).

**Table 1:** Characteristic of included patients

	Non-IPM	IPM	*p* value
*n*	74	108	
Age (year)	49.15 ± 11.10	51.45 ± 12.45	0.202
Sex (male/female)	63/11	87/21	0.425
Liver-related basic disease			
Cirrhosis	26	40	0.793
Portal hypertension	10	13	0.768
Hypersplenism	11	14	0.714
HbsAg(+)	64	83	0.105
HBV DNA(+)	48	52	0.07
AFP > 40	36	55	0.926
Others	16	17	0.312
Non-liver combined disease			
Hypertension	6	5	0.333
Diabetes	8	14	0.662
Others	14	16	0.464
Liver injury			
ALT (U/L)	40 (24–75)	36.6(22–64)	0.5674
AST (U/L)	44 (29–70)	45(30.6–75)	0.9066
Liver function reserve			
Albumin (g/L)	41.4 (38.6–44.8)	41.9 (38.1–45.2)	0.944
Total bilirubin (μmol/L)	16.5 (13.3–20.4)	15.8 (12.75–22.5)	0.9965
Child-Pugh classification			
5	60	91	
6	5	4	
7	4	6	
8	2	5	0.721
Coagulation function			
Prothrombin time (seconds)	11.60 (11.1–12.3)	11.7 (11–12.4)	0.6764
Platelet(× 10^9^/L)	165 (123–202)	185 (128–248)	0.0683
Characteristics of hepatic carcinoma patients			
TNM stage			
1	42	63	
2	13	18	
3	12	20	
4	6	6	0.954
Cancer embolus	20	25	0.551
Lymphatic metastasis	3	6	0.904

*Abbreviations*: *AFP* alpha-fetoprotein, *ALT* alanine transaminase, *AST* aspartate aminotransferase, *IPM* intermittent Pringle maneuver, *TNM stage* tumor, lymph node, metastasis stage

The operative time and bleeding volume in the IPM group were not significantly different from those in the non-IPM group. No statistical differences in the extent of hepatectomy and the number of injured hepatic segments were found between the two groups. The duration of portal occlusion was 50 (30–80) min, and the number of occlusions was 5 (3–8) in the IPM group. No differences were found between the two groups in terms of the performance of cholecystectomy (*p* = 0.075) and other accessory procedures (*p* = 0.312) (Table 2).

**Table 2:** Characteristic of patients during and after surgery

	Non IPM	IPM	*p* value
Surgery time (min)	300 (245–390)	288.5 (219–356)	0.187
Amount of bleeding (ml)	500 (300–800)	400 (200–800)	0.0941
Hepatic portal occlusion time (min)	0	50 (30–80)	NA
Count of occlusion	0	5 (3–8)	NA
Type of operation			
Partial lobectomy	44	59	
Left lobectomy	9	16	
Right lobectomy	18	28	
Trisegmentectomy	3	5	0.922
Number of injured hepatic segments			
1	6	17	
2	27	27	
3	16	27	
4	22	31	
5	3	6	0.354
Attach surgery			
Cholecystectomy	54	65	0.075
Others	16	17	0.312
Postoperative complication			
Bleeding	0	2	0.239
Biliary fistula	2	0	0.086
Incision infection	1	1	0.787
Intra-abdominal abscess	1	2	0.794
Sectional effusion	4	9	0.451
*Ascites*	22	17	0.024
Pulmonary infection	27	27	0.096
*Pleural effusion*	48	53	0.035
Respiratory failure	2	0	0.086
Liver failure/dysfunction	0	0	NA
Death	0	2	0.239
Hospital duration (day)	15 (12–17)	12 (10–16)	0.0008
ICU duration (day)	3 (2–3)	2 (2–3)	0.1478

*Abbreviations*: *ICU* intensive care unit, *IPM* intermittent Pringle maneuver, *NA* not available

In terms of postoperative complications, no significant differences in postoperative bleeding, bile leakage, incision infection, abdominal abscess, incision effusion, pulmonary infection, hepatic insufficiency/liver failure, or death were found between the two groups. The incidence rates of ascites (*p* = 0.024) and pleural effusion (*p* = 0.035) in the IPM group were significantly lower than those in the non-IPM group. The postoperative hospitalization time in the IPM group was significantly shorter than that in the non-IPM group (*p* = 0.0008) (Table 2). Two patients died in the IPM group; the causes of death were organ failure after abdominal infection and cardiac arrest due to cardiac insufficiency. However, no difference in the incidence of abdominal infection or incision infection was found between the two groups.

On the first day after the operation, the leukocyte count, neutrophil ratio, and ALT, AST, albumin, total bilirubin, and D-D levels were not significantly different between the two groups. The platelet count in the non-IPM group was significantly lower than that in the IPM group (*p* = 0.0381), but the PT (*p* = 0.0195) and APTT (*p* = 0.0071) in the non-IPM group were significantly higher than those in the IPM group. On the third day after the operation, only the neutrophil ratio remained significantly higher in the non-IPM group than that in the IPM group (*p* = 0.0057), and no significant differences were found in the other indexes. On the fifth and seventh postoperative days, no significant differences in any of the indexes were observed between the two groups. At discharge, only albumin in the non-IPM group was significantly higher than that in the IPM group (*p* = 0.0303) (Table 3). The statistically different results did not change after correction by the FDR method. The length of hospitalization showed the most pronounced difference between the non-IPM and IPM groups (*p* = 0.0008), followed by the third-day neutral cell ratio (*p* = 0.0057) and the first-day APTT (*p* = 0.0071). Importantly, no significant differences in ALT and AST levels were noted between the two groups.

**Table 3:** Results of blood biochemistry and coagulation function after operation

	Non IPM	IPM	*p* value
First day after operation			
WBC	11.38 (9.05–14.08)	12.15 (9.85–15.3)	0.2386
NLR	88.08 ± 3.68	87.33 ± 4.12	0.2307
PLT	132 (105–168)	152 (114–210)	0.0381
ALT	242.5 (129–336)	157.1 (98.2–384.3)	0.1709
AST	254 (171–418)	208.8 (126.05–400.55)	0.1148
ALB	32.48 ± 5.11	32.24 ± 6.05	0.7739
TB	25.8 (17.5–40.9)	22.4 (15.7–33)	0.07
PT	15.3 (14.3–17)	14.3 (13.5–15.8)	0.0195
APTT	36.3 (32–43.4)	33.2 (28.6–38.7)	0.0071
D-Dimer	2.92 (0.83–4.46)	3.765 (2.06–4.85)	0.1478
Third day after operation			
WBC	8.43 (5.78–10.59)	8.39 (6.47–10.84)	0.7004
NLR	83.51 ± 5.3	80.5 ± 5.91	0.0057
PLT	106.5 (74–158)	126 (100–167)	0.075
ALT	133.9 (92–229.8)	104.1 (71–245)	0.2797
AST	86 (67–132)	89 (64–140.4)	0.7739
ALB	37.84 ± 5.09	37.08 ± 5.43	0.4332
TB	22.7 (15.5–37.5)	26.05 (17.4–34.7)	0.6339
PT	15.8 ± 3	14.9 ± 2.4	0.2071
APTT	40.8 (33.4–52.4)	38.15 (32.6–42.9)	0.1275
D-Dimer	3.84 (0.893–6.38)	4.65 (2.8–9.1)	0.1136
Fifth day after operation			
WBC	7.89 ± 3.05	8.05 ± 2.81	0.8301
NLR	75.37 ± 8.86	72.98 ± 6.47	0.2143
PLT	110.5 (72–176)	148 (119–198)	0.0739
ALT	74.5 (53–104)	87 (59–139)	0.3716
AST	50 (35–62)	52.1 (33–71)	0.5623
ALB	36.2 ± 4.35	37.57 ± 4.24	0.2176
TB	24.05 (13.7–32.8)	25.6 (18.4–33.6)	0.807
PT	15.8 (14.05–17.65)	14.8 (13.1–16)	0.2038
APTT	40.85 (34.35–48.95)	37.4 (30.9–38.8)	0.0526
D-Dimer	5.5 (1.15–9.53)	9.62 (1.08–14.2)	0.3496
Seventh day after operation			
WBC	7.69 (7.08–11.19)	8.28 (7.39–12.04)	0.529
NLR	70.89 ± 7.01	69.82 ± 8.18	0.6239
PLT	178.11 ± 56.26	192.69 ± 98.42	0.5614
ALT	49 (44–80)	55 (42–100)	0.5803
AST	32 (23–49)	38.1 (29–53.9)	0.2185
ALB	36.23 ± 4.37	35.91 ± 3.52	0.7621
TB	19.5 (15.1–51.7)	21.1 (16.1–32.3)	0.9008
PT	14.45 (13.6–17.25)	15 (13.6–16.6)	0.9422
APTT	35.8 (29.7–46.85)	32.2 (29.7–61)	0.8283
D-Dimer	5.715 (1.42–13.31)	7.41 (0.748–10.93)	0.665
Discharge from hospital			
WBC	6.35 ± 2.66	6.49 ± 2.27	0.7368
NLR	67.56 ± 9.15	67.09 ± 7.5	0.7466
PLT	184.5 (128–249)	178 (126–223)	0.5397
ALT	46 (35–61)	48.8 (32.1–65.9)	0.2943
AST	35 (25–46)	36.6 (28.1–51)	0.2819
ALB	37.5 (33–40.8)	35.5 (31.9–37.9)	0.0303
TB	16.4 (13–25.5)	16.7(11.9–25.2)	0.8516
PT	13.05 (12.2–14.9)	13.1(12.2–14.1)	0.9695
APTT	28.8 (27.4–34.8)	30.45 (28.5–35)	0.3497
D-Dimer	8.67 ± 5.91	9.54 ± 5.14	0.7053

*Abbreviations*: *WBC* white blood cell, *NLR* neutrophil to lymphocyte ratio, *PLT* platelet, *ALT* alanine transaminase, *AST* aspartate transaminase, *ALB* albumin, *TB* total bilirubin, *PT* prothrombin time, *APTT* activated partial thrombin time

Footnote: Normal distribution quantitative data is expressed as mean ± standard deviation and compare the group comparison using analysis of variance; non-distribution data is expressed as median and quartile and compare the group comparison using Kruskal-Wallis test

The effects of hepatic interruption time on the levels of ALT and AST after surgery were analyzed by regression (Table 4). Univariate regression showed that a prolonged interruption time was related to increased levels of ALT (coef = 1.66, *p* = 0.001) and AST (coef = 2.00, *p* = 0.002) on the first day after the operation. On the fifth day after the operation, the interruption time was significantly correlated with the level of ALT (coef = 0.41, *p* = 0.038). No correlation was found between the interruption time and the levels of ALT and AST on the seventh day after the operation or at discharge. Multivariate analysis yielded similar results.

**Table 4:** Univariate and multivariate analysis of hepatic vascular occlusion time and postoperative ALT AST results

Hepatocellular carcinoma patients		Univariate analysis		Multivariate analysis#	
After operation	Index	Coef. (95% CI)	*p* value	Coef. (95% CI)	*p*
1st day	ALT	1.66 (0.67, 2.65)	0.001	1.61 (0.6, 2.63)	0.002
	AST	2 (0.76, 3.24)	0.002	1.8 (0.5, 3.1)	0.007
3rd day	ALT	0.36 (− 0.37, 1.09)	0.332	0.42 (− 0.34, 1.18)	0.277
	AST	0.24 (− 0.06, 0.54)	0.118	0.26 (− 0.04, 0.55)	0.088
5th day	ALT	0.41 (0.02, 0.8)	0.038	0.41 (0.01, 0.81)	0.043
	AST	NA		0.11 (− 0.04, 0.26)	0.140
7th day	ALT	NA		NA	NA
	AST	NA		NA	NA
Discharge	ALT	0.08 (− 0.08, 0.24)	0.315	0.1 (− 0.06, 0.26)	0.213
	AST	− 0.01 (− 0.13, 0.1)	0.817	− 0.04 (− 0.17, 0.08)	0.526

#Adjust for age, sex, and preoperative ALT/AST variants

**Fig. 1:**
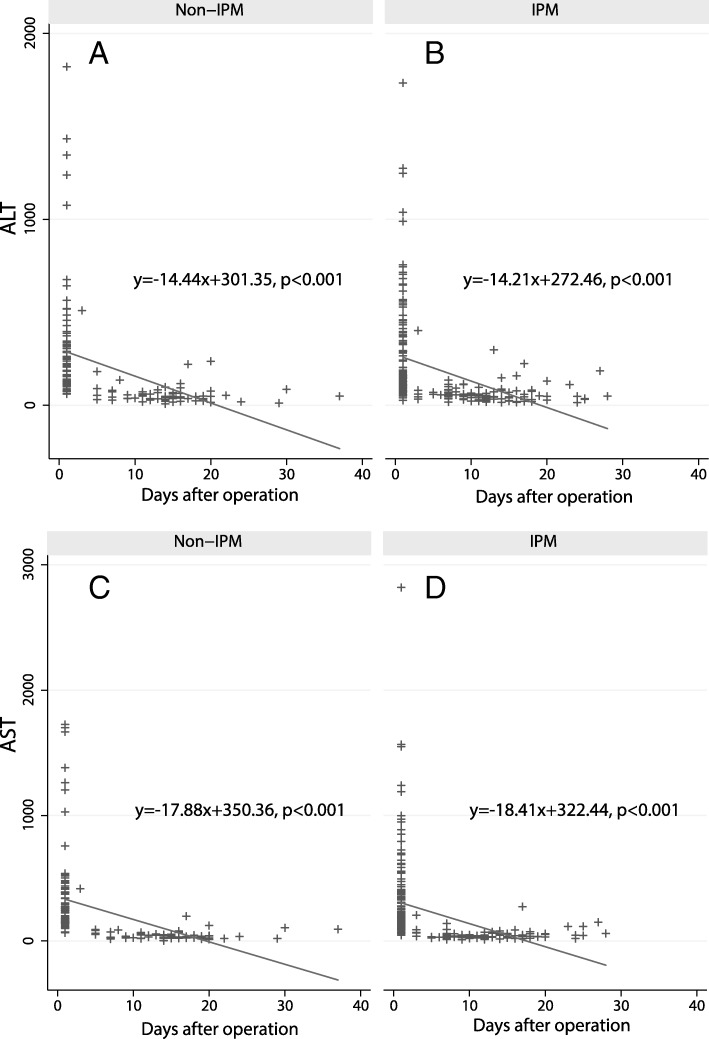
Regression analysis of ALT/AST recovery with postoperative time in non-IPM and IPM patients. **a** ALT changes in non-IPM patients. **b** ALT changes in IPM patients. **c** AST changes in non-IPM patients. **d** AST changes in IPM patients

Regression analysis was used to analyze the relationships between the levels of ALT and AST and postoperative hospitalization time in the IPM group and non-IPM group. The regression relationships between the levels of ALT (*y* = − 14.44*x* + 301.35, *p* < 0.001) and AST (*y* = − 17.88*x* + 350.36, *p* < 0.001) and postoperative time were significant in the non-IPM group (Fig. 1a, c). The regression relationships between the levels of ALT (*y* = − 14.21*x* + 272.46, *p* < 0.001) and AST (*y* = − 18.41*x* + 322.44, *p* < 0.001) and postoperative time were also significant in the IPM group (Fig. 1b, d). No significant difference in the regression coefficients was found between the two groups (ALT: *p* = 0.9387; AST: *p* = 0.8901).

## Discussion

This study retrospectively analyzed 182 hepatocellular carcinoma patients who underwent hepatectomy and evaluated the effect of the IPM on the postoperative hepatocellular injury. In our study, the incidence rates of pleural effusion and ascites were higher in the non-IPM group than those in the IPM group. The hospitalization time in the IPM group was clearly shorter than that in the non-IPM group. The platelet count in the non-IPM group was significantly lower than that in the IPM group. The PT and APTT in the non-IPM group were significantly higher than those in the IPM group on the first day after surgery. On the third day after the operation, the neutrophil ratio in the non-IPM group was significantly higher than that in the IPM group. At discharge, only albumin in the non-IPM group was significantly higher than that in the IPM group. Other indicators showed no significant differences between the two groups. In the regression analysis of the levels of ALT and AST and the total hepatic interruption time, the ALT and AST levels on the first day after surgery increased with prolongation of the interruption time.

In this study, we found that the PT and APTT in the non-IPM group were significantly higher than those in the IPM group, and the platelet count was lower in the non-IPM group. More blood loss was recorded in the non-IPM group than that in the IPM group, but the difference was not statistically significant (*p* = 0.0941), which may explain the increased PT and APTT in the non-IPM group. The difference disappeared on the third day after surgery. A prospective study also suggested that the PM can reduce bleeding during hepatectomy, minimize hemodynamic disturbances, and protect liver function in the early postoperative period [12]. The PM is even considered safe for patients with severe cirrhosis [6]. In addition, intermittent occlusion of the hepatic hilum may result in hepatic tissue tolerance of and protection against ischemia-reperfusion injury [7]. Liver ischemia preconditioning before the PM is applied has also been shown to enhance liver tolerance [13]. In liver transplantation, intermittent blood flow interruption has no significant effect on liver function and injury [14, 15]. Our study also indicated that hepatic vascular occlusion had no significant effect on liver injury and liver function.

This study compared hepatectomies with and without the IPM. Meanwhile, controversy remains regarding the use of continuous and intermittent PMs. A study suggested that continuous PMs can more successfully reduce liver injury and promote liver recovery than IPMs [16]. However, another study reported no significant difference in liver injury between patients undergoing continuous and intermittent interruption of the hepatic blood flow [17]. This finding may be related to the duration of the interruption time. If the duration of a single interruption event does not exceed the threshold for liver ischemia-reperfusion injury, then liver damage will not occur. Once the threshold is exceeded, however, the blood flow interruption will cause liver damage. Therefore, the effect of the duration of a single interruption event on postoperative liver recovery may exceed that of the total interruption time.

In a clinical prospective randomized controlled trial (RCT), the performance of the IPM with intervals of 30 min was considered safe [18]. In other retrospective clinical studies, the authors concluded that the IPM with clamping times exceeding 60–120 min was still safe [19–21]. In this study, more intensive circulation was achieved using an intermittent strategy with cycles of 10 min of inflow occlusion followed by 5 min of reperfusion. This intermittent strategy did not cause significant liver damage in this study. Therefore, the interruption strategy should be clearly stated in future reports on the IPM to allow comparisons among studies.

Regarding recovery from hepatocellular injury after surgery, the levels of ALT and AST decreased gradually as the postoperative time increased in both the IPM and non-IPM groups, and no significant difference in postoperative liver injury was noted between the two groups in this study. In terms of postoperative complications, this study found higher incidence rates of ascites and pleural effusion in the non-IPM group than those in the IPM group. In an RCT with patients with liver tumors, the population receiving the IPM had higher rates of subclinical ascites and pleural effusion than the non-IPM population [22] in contrast to our reported results; however, the ascites and pleural effusion rates were determined based on radiological measurements rather than clinical testing. In addition, this study also concluded that the IPM had no significant effect on the length of hospitalization after surgery [22].

A recent retrospective study analyzed hepatectomy patients in the liver-targeted National Surgical Quality Improvement Program (NSQIP) database (2014–2016), but the heterogeneity of this study was relatively high [23]. The study indicated that the PM was associated with a longer total hospital length of stay based on a comparison with non-PM cases. Other studies have reported no difference in the incidence of post-hepatectomy liver failure or the need for blood transfusion. Our study further defined IPM strategies and hepatocellular carcinoma patients undergoing hepatectomy and revealed that the IPM does not cause additional liver damage. No significant difference in intraoperative blood loss was observed between the study groups. We believe that the IPM can facilitate better surgical visualization and concluded that application of the PM results in a shorter hospitalization time based on this study, but this outcome may be related to the characteristics of various patient populations or even to the policies of local hospitals.

Finally, whether ischemia-reperfusion injury caused by the PM can promote the recurrence and metastasis of hepatic tumors and affect patient prognosis remains controversial in the clinical setting. Research results suggest that the IPM is safe for patients with liver cancer [24–26]. This study mainly analyzed the role of the IPM in liver damage and liver injury after hepatic surgery, and long-term follow-up results are required to determine the impact of the IPM on liver cancer patients.

## Conclusions

This study concluded that the IPM does not cause additional liver damage during hepatectomy. In addition, the use of the IPM results in shorter hospital stays compared to surgery without using the IPM. However, the results of this study require further confirmation because of the retrospective design.

## Additional files

Additional file 1: Illustration of surgical procedure. (DOCX 3149 kb)

## Data Availability

All data generated or analyzed during this study are included in this published article.

## Abbreviations

AFP: Alpha-fetoprotein
ALT: Alanine aminotransferase
ANOVA: Analysis of variance
APTT: Activated partial thromboplastin time
AST: Aspartate aminotransferase
D-D: D-dimer
DNA: Deoxyribonucleic acid
HBsAg: Hepatitis B surface antigen
HBV: Hepatitis B virus
ICU: Intensive care unit
IPM: Intermittent Pringle maneuver
PM: Pringle maneuver
PT: Prothrombin time
RCT: Randomized controlled trial
TNM: Tumor, node, metastasis
