# Supplementary material for: Effect of the intermittent Pringle maneuver on liver damage after hepatectomy: a retrospective cohort study
Source: World J Surg Oncol. 2019 Aug 13;17:142. doi: 10.1186/s12957-019-1680-y (PMC6693131; doi:10.1186/s12957-019-1680-y)
Supplement: Supplementary file 2 — Illustration of surgical procedure. (DOCX 3149 kb) [file 12957_2019_1680_MOESM1_ESM.docx]

Surgical procedure


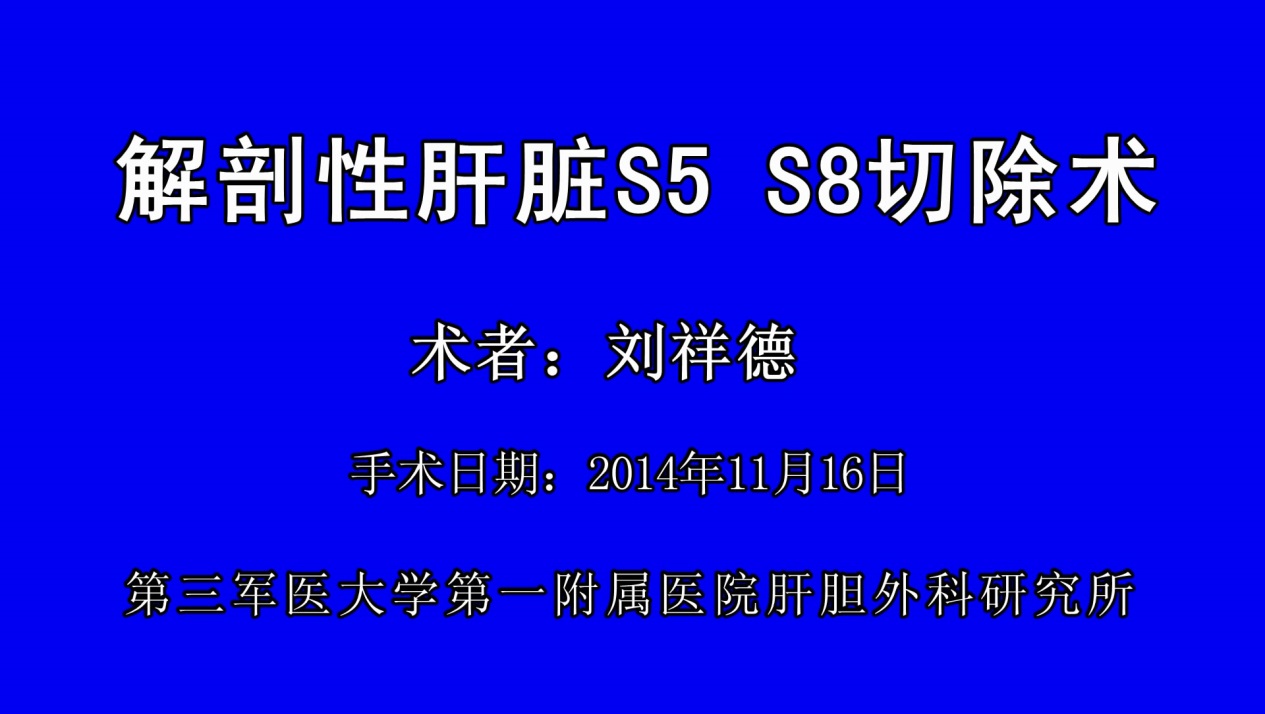


Anatomical hepatectomy(S5, S8)

Surgeon: Xiangde Liu

Date: 2014-11-16

Address: Institute of Hepatobiliary Surgery, First affiliated hospital of Third Military Medical University (Army Medical University).


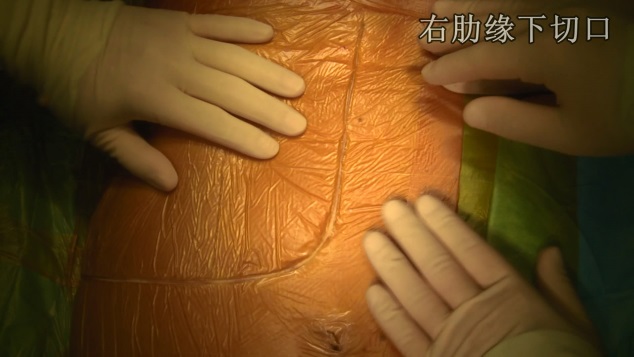

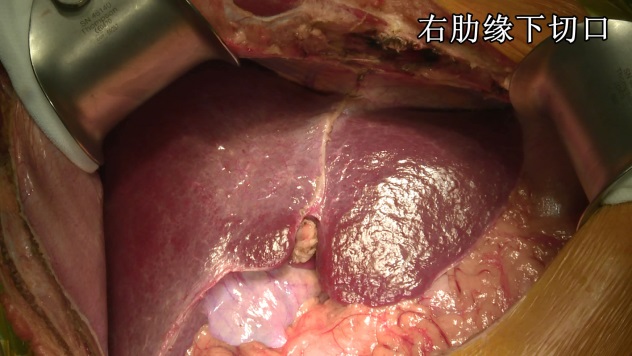


Right subcostal incision


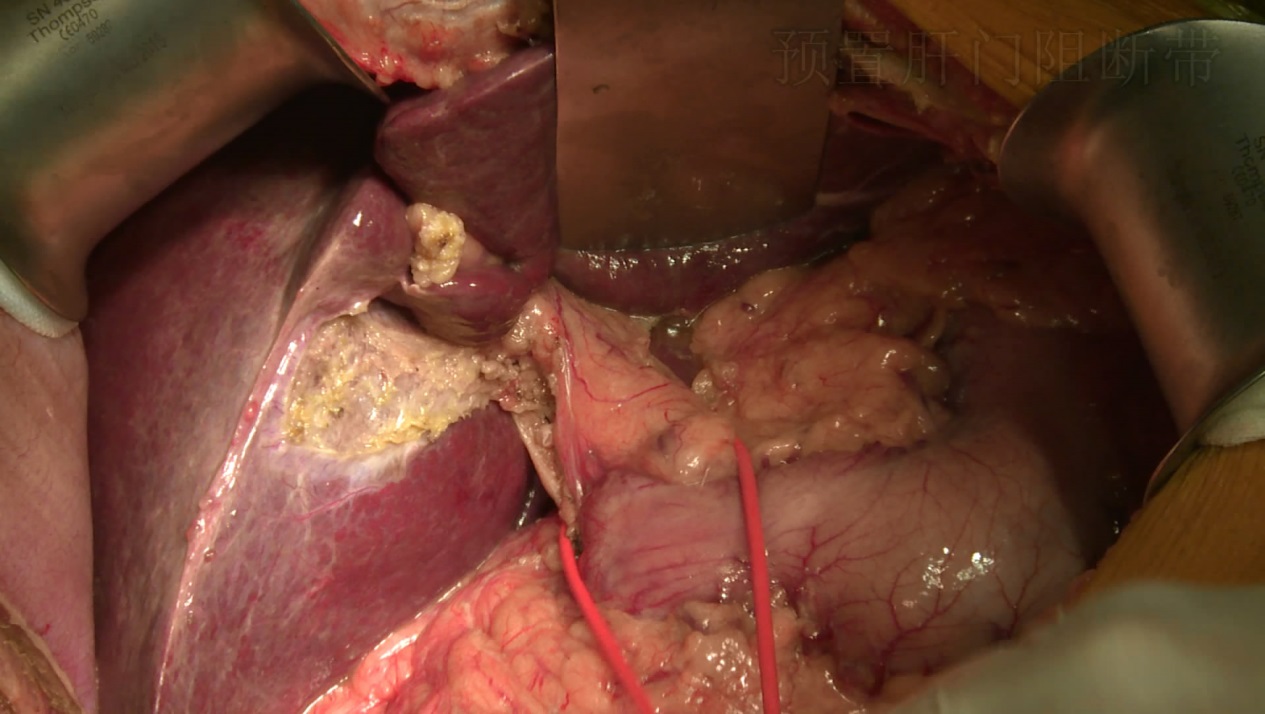


Preset hepatic portal occlusion


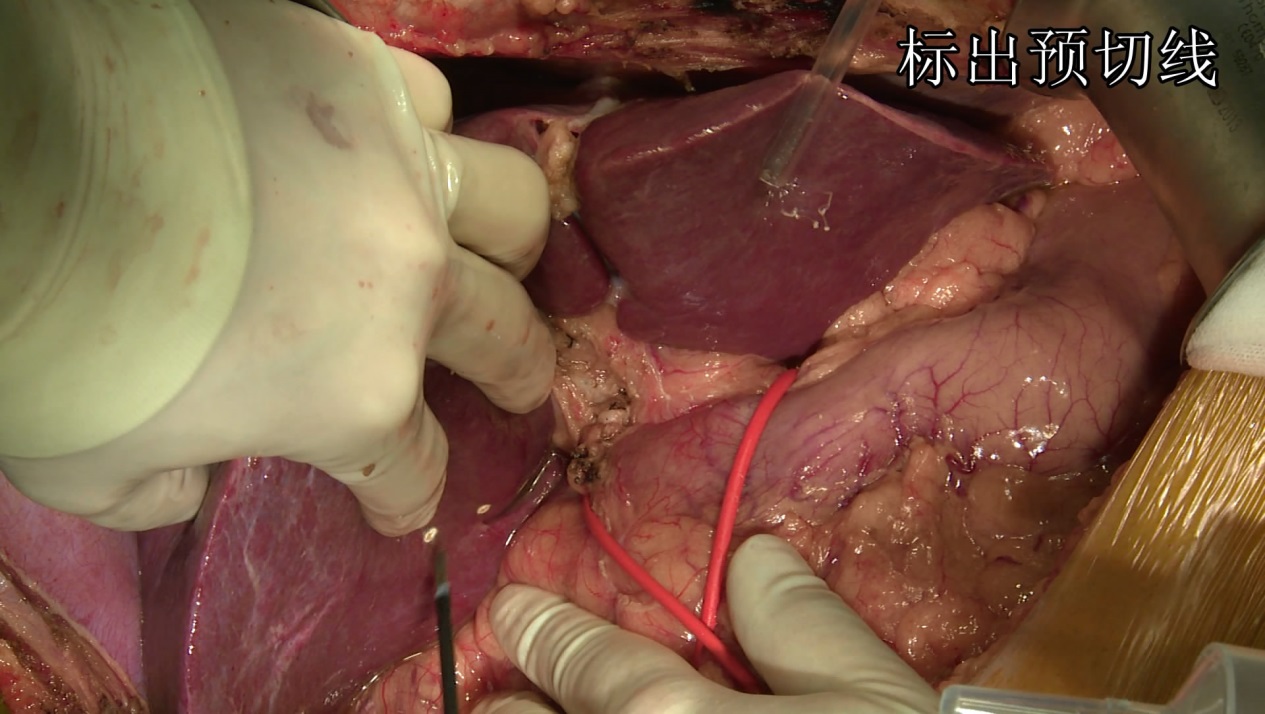


Mark line of resection


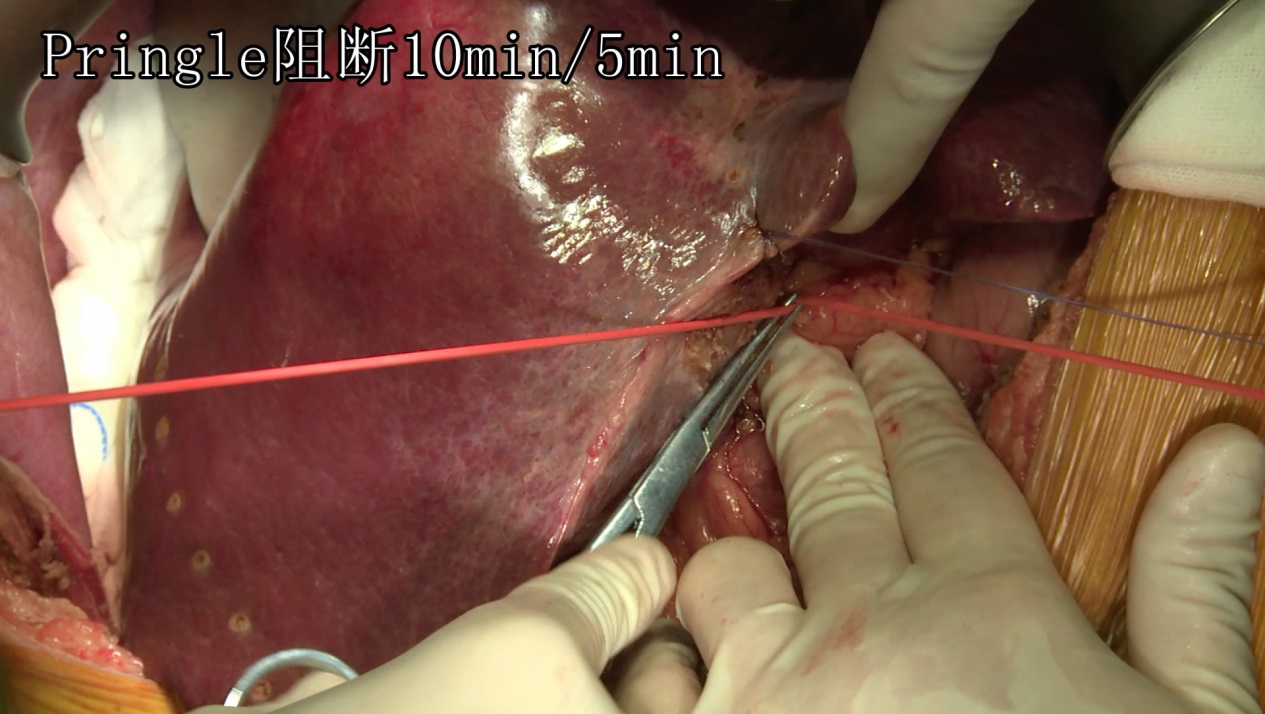


Pringle occlusion: 10 minutes of inflow occlusion followed by 5 minutes of reperfusion


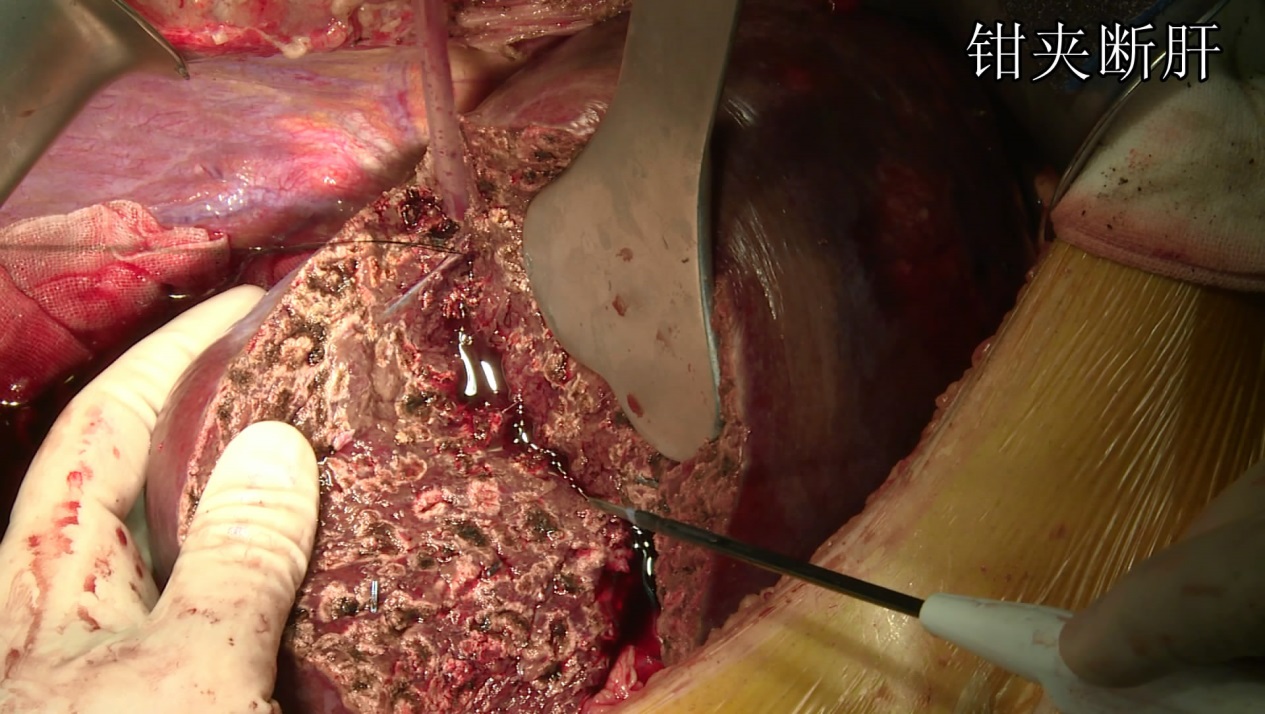


Clump Crushing Technique


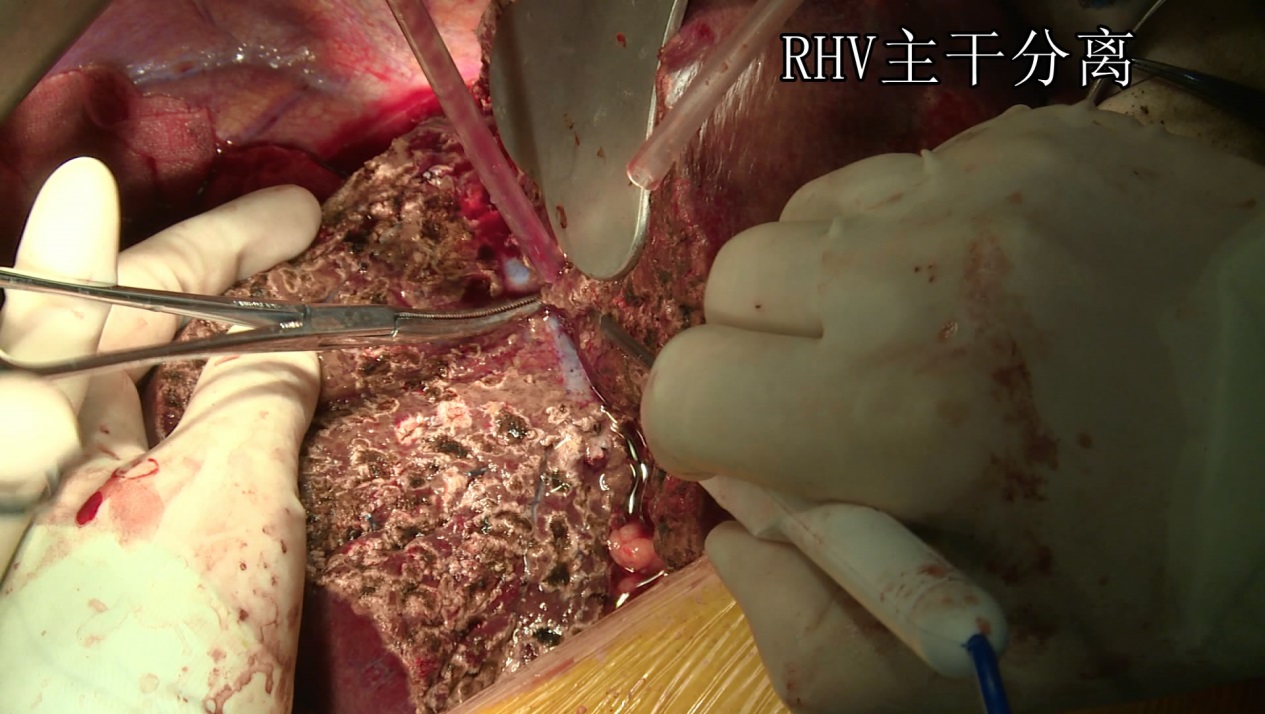


Isolation of Right Hepatic Vein (RHV) trunk


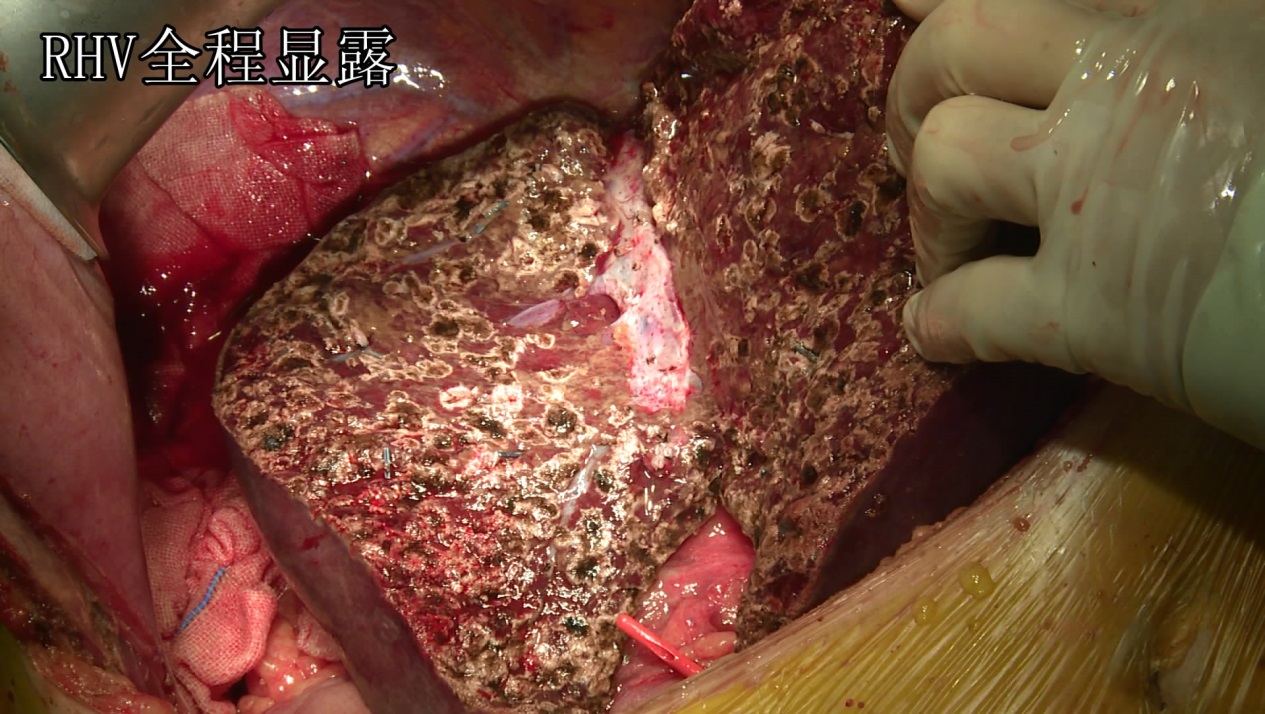


RHV exposure


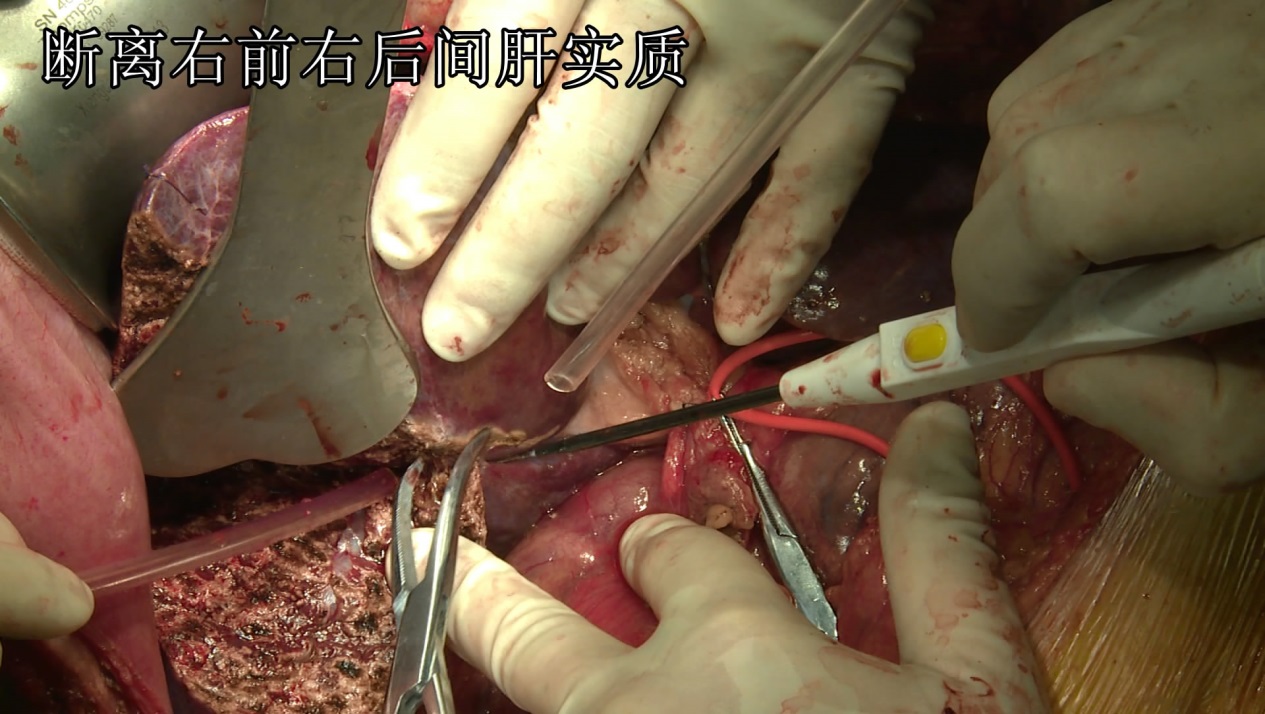


Separation of right anterior and right posterior hepatic parenchyma


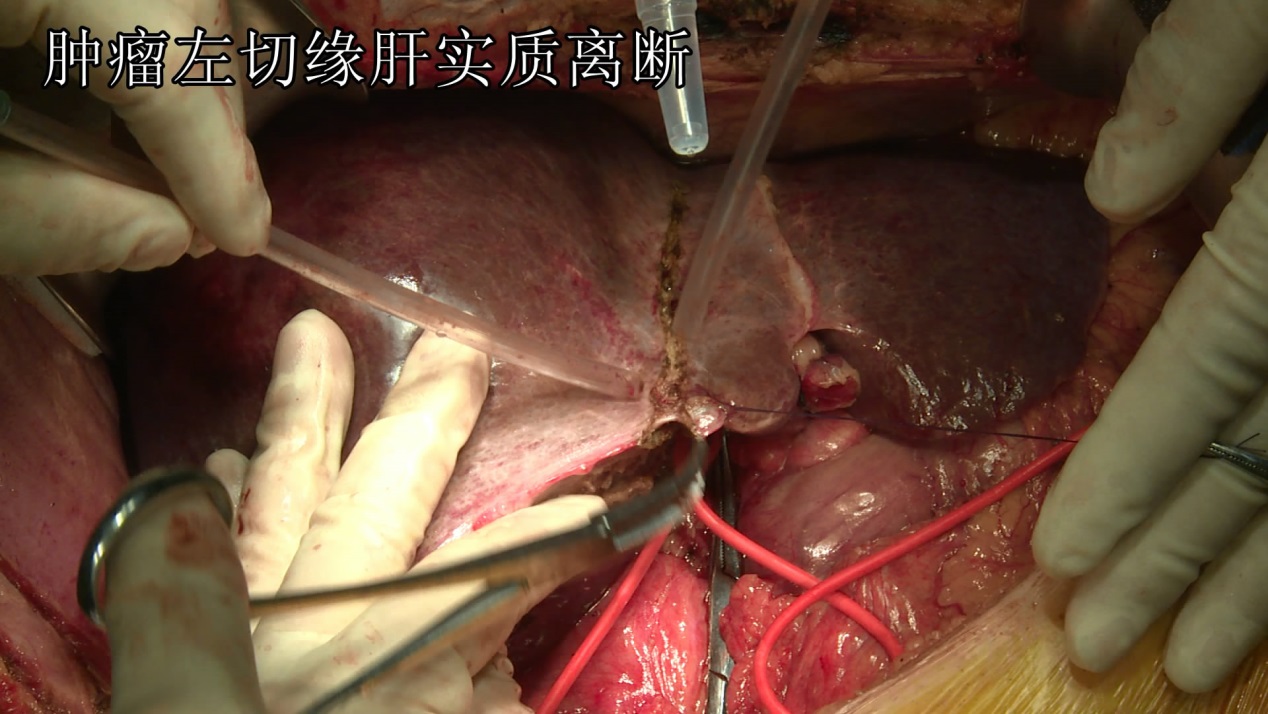


Separation of tumor left resection margin


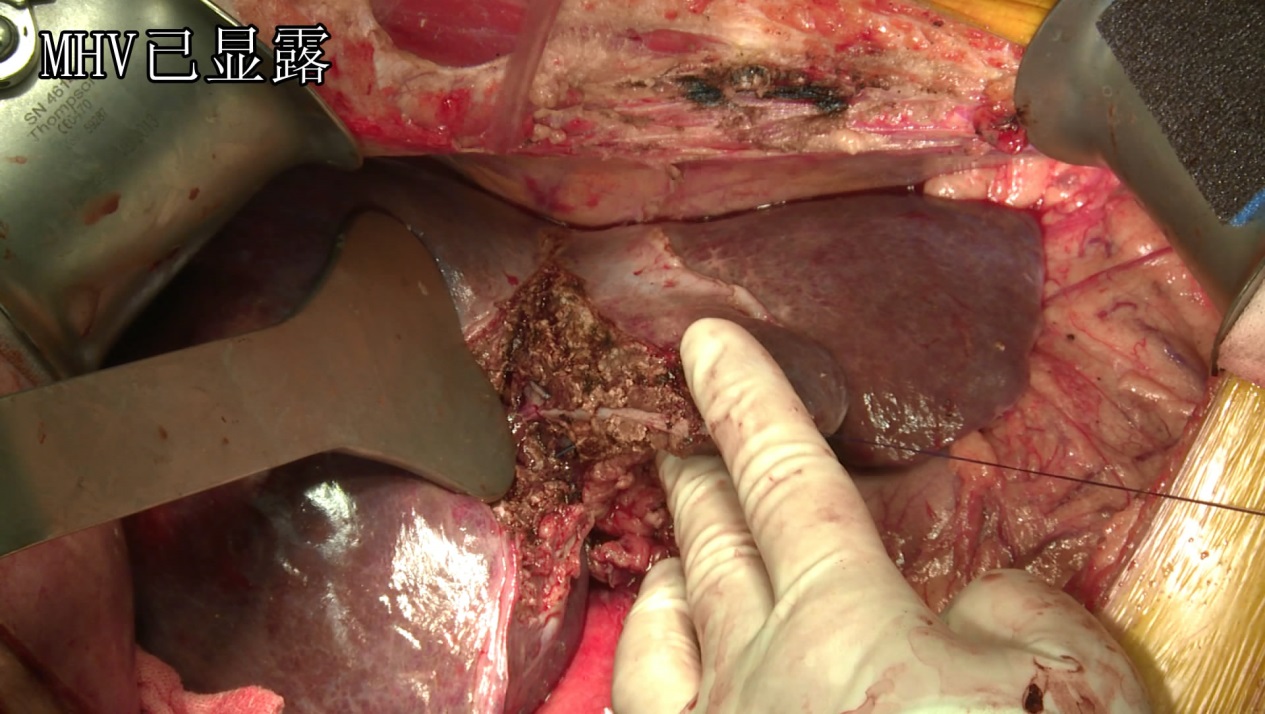


Middle hepatic vein (MHV) exposure


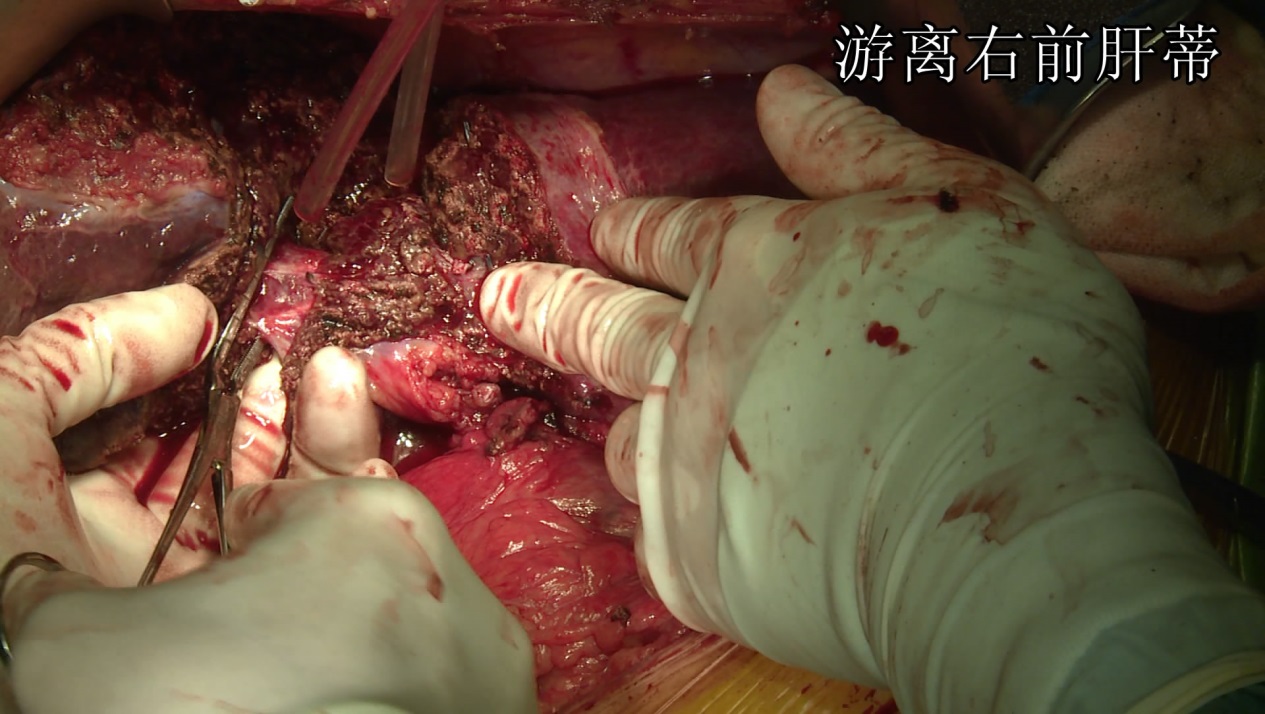


Isolation of right anterior hepatic pedicle


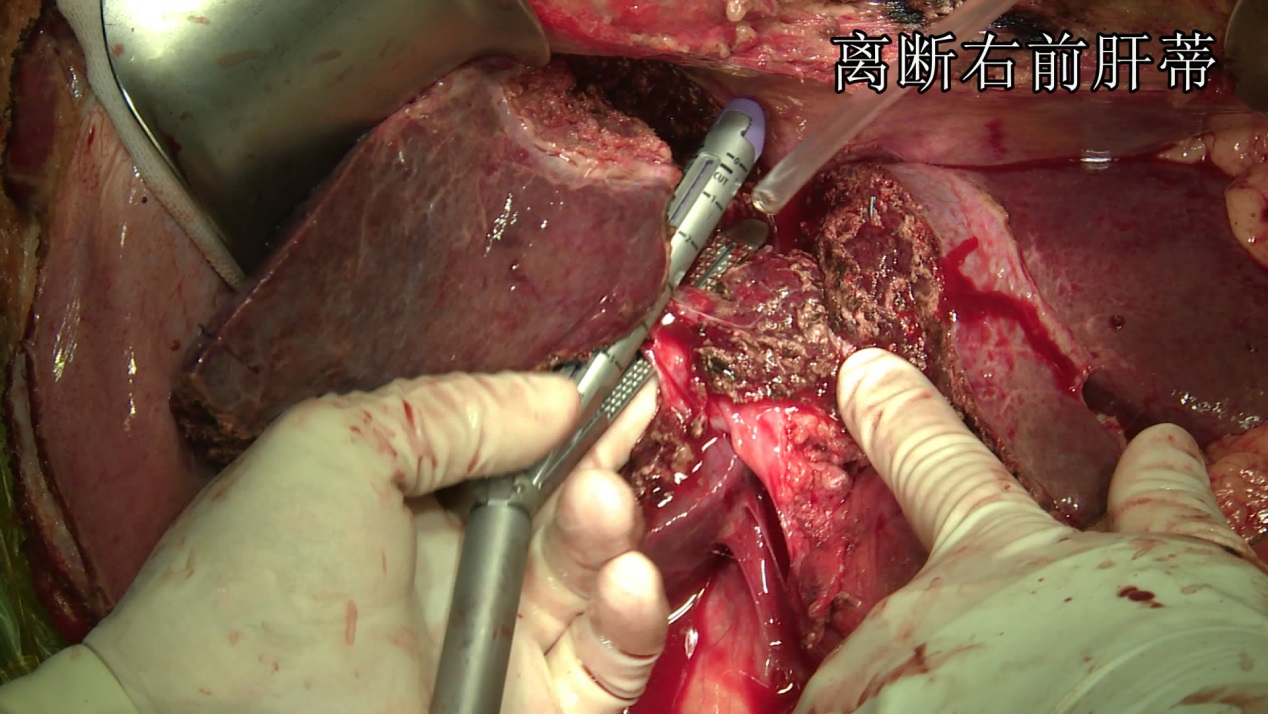


Cut off right anterior hepatic pedicle


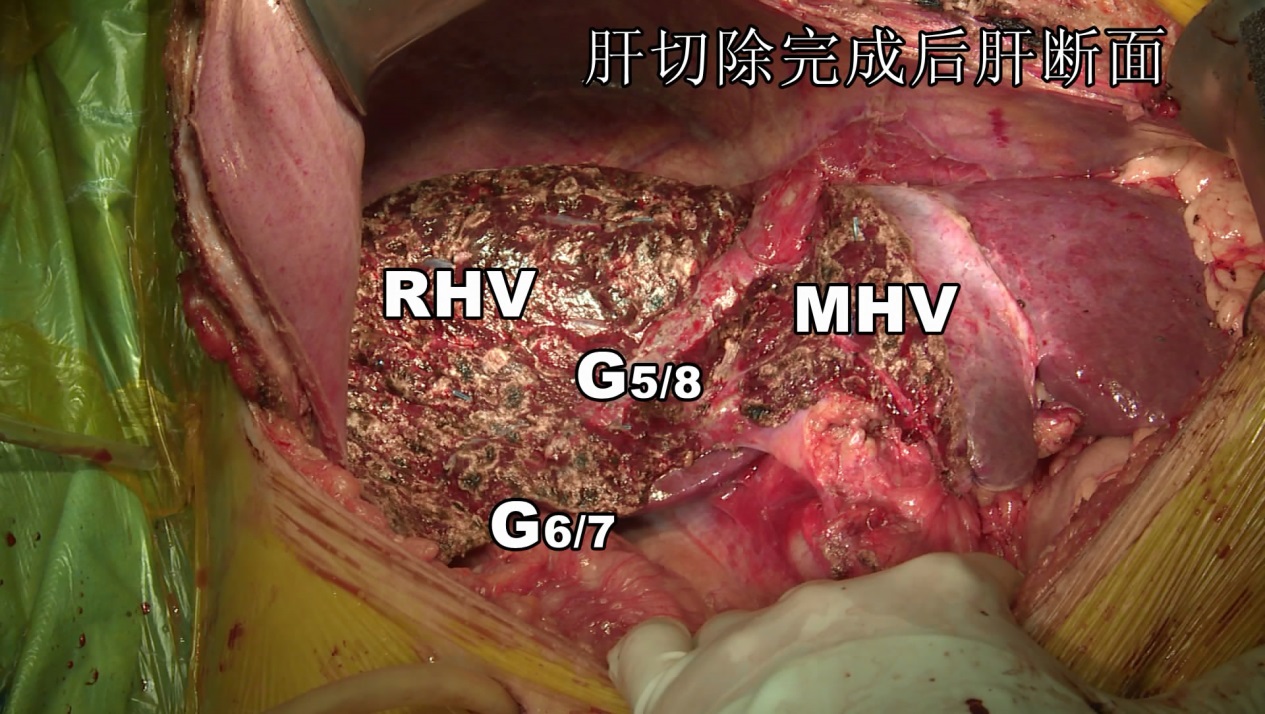


Section after hepatectomy


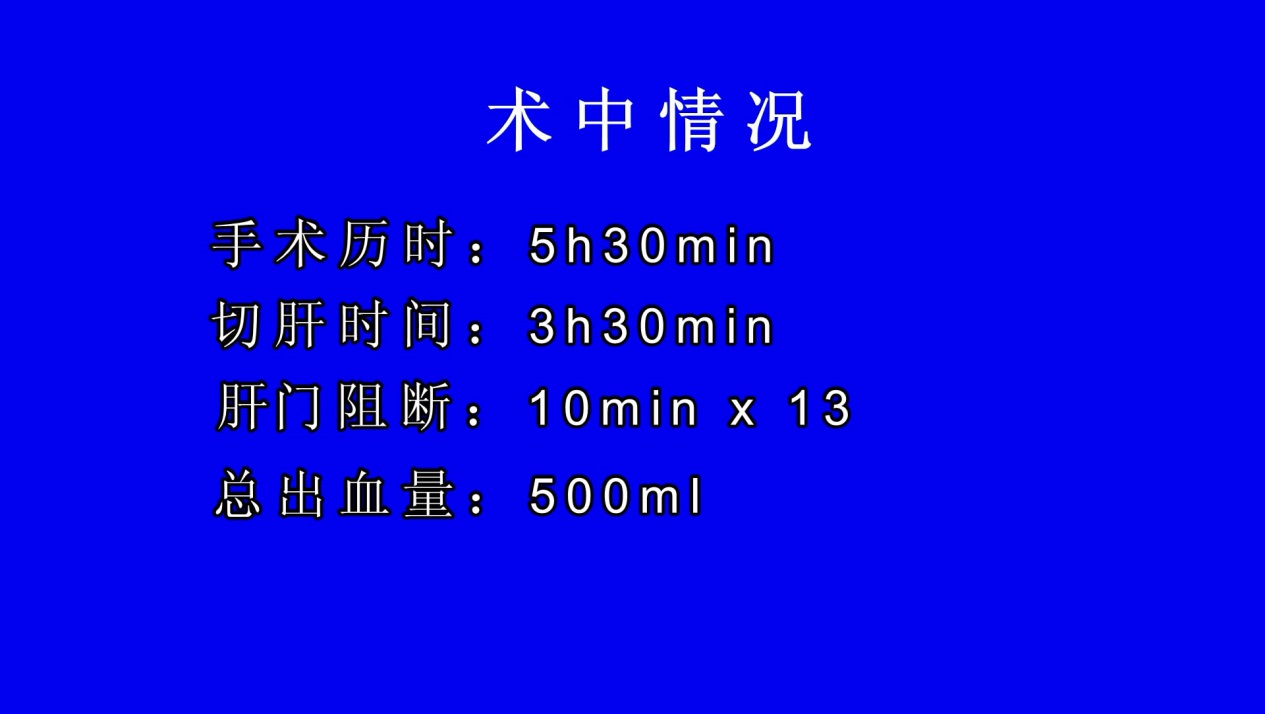


Intraoperative conditions

Duration of operation: 5 hours and 30 minutes

Duration of liver resection: 3 hours and 30 minutes

Duration of hepatic vascular occlusion: 10 minutes × 13

Amount of bleeding: 500ml
